# Changes in monocyte subsets are associated with an increased risk of AAA and are surrogate markers for AAA morphology in patients with late-stage disease

**DOI:** 10.3389/fimmu.2025.1621888

**Published:** 2025-09-03

**Authors:** Bianca Hamann, Anna Klimova, Marvin Kapalla, David M. Poitz, Albert Busch, Henning Morawietz, Christian Reeps, Anja Hofmann

**Affiliations:** ^1^ Division of Vascular and Endovascular Surgery, Department of Visceral, Thoracic and Vascular Surger, Faculty of Medicine and University Hospital Carl Gustav Carus, TUD Dresden University of Technology, Dresden, Germany; ^2^ Institute for Medical Informatics and Biometry, Faculty of Medicine, TUD Dresden University of Technology, Dresden, Germany; ^3^ Institute for Clinical Chemistry and Laboratory Medicine; University Hospital and Medical Faculty Carl Gustav Carus, TUD Dresden University of Technology, Dresden, Germany; ^4^ Division of Vascular Endothelium and Microcirculation, Department of Medicine III, University Hospital and Medical Faculty Carl Gustav Carus, TUD Dresden University of Technology, Dresden, Germany

**Keywords:** AAA, monocyte subsets, biomarker, cardiovascular, surrogate marker

## Abstract

**Introduction:**

Monocytes play a role in the pathology of abdominal aortic aneurysm (AAA) and can display immunophenotypic heterogeneity. Alterations in monocyte subsets are associated with cardiovascular risk, but their profile in AAA is poorly understood.

**Aim:**

We aimed to comprehensively define associations of monocyte phenotypes with AAA risk and AAA morphology.

**Methods:**

Monocyte subsets (CD14++CD16−, CD14++/CD16+, and CD14+/CD16++) were analyzed in an observational study in patients with AAA (n = 33) and varicose veins (n = 33) using flow cytometry.

**Results:**

Classical monocytes were 3% lower (*p* = 0.001) in AAA, while intermediate and non-classical monocytes were 1.8-fold (*p* = 0.019) and 1.9-fold (*p* = 0.025) higher in AAA, respectively. The differences remained significant after adjusting for age, sex, and peripheral artery disease. A decrease in classical monocytes [odds ratio (OR): 0.73, *p* = 0.002] and increases in intermediate (OR: 1.41, *p* = 0.006) and non-classical monocytes (OR: 1.54, *p* = 0.030) were associated with a higher risk of AAA. Non-classical monocytes showed an inverse correlation with AAA diameter (r_P_ = −0.64, *p* = 0.001) and AAA volume (r_P_ = −0.50, *p* = 0.003).

**Conclusion:**

The present study revealed age- and sex-independent shifts in monocytes, all of which were associated with the risk of AAA disease. Non-classical monocytes were inversely correlated with AAA diameter and volume and thus may be surrogate markers for AAA morphology.

## 1 Introduction

Abdominal aortic aneurysm (AAA) is a progressive pathological dilatation of the abdominal aorta, and eventual AAA rupture is a significant cause of death in elderly people (1). Currently, exclusion (open or endovascular repair) is the only effective treatment, and the decision for elective treatment is based solely on the maximum diameter. Surgery is recommended once the aortic diameter exceeds >55 mm in men and 50 mm in women. However, individual differences in AAA wall remodeling (2) and individual growth rates (1) result in different risks of AAA rupture. Moreover, small AAAs are also susceptible to rupture (1). This highlights the urgent need to develop novel therapeutic and diagnostic strategies to improve clinical management and decision making (3). Ideally, a circulating biomarker would be involved in the pathogenesis of AAA, reflecting aortic wall remodeling or inflammatory activity (4).

The main pathological features of AAA are inflammation, extracellular matrix degradation, and phenotypic changes in smooth muscle cells (5). Inflammatory cells in AAA walls comprise monocytes, macrophages, neutrophils, mast cells, natural killer (NK) cells, T and B cells, and dendritic cells (6). Monocytes are recruited to sites of inflammation, where they differentiate into macrophages (7). Monocytes exist in subsets that differ in terms of their morphology, function, and expression of cell surface markers. Three distinct subsets were distinguished in humans based on the expression of the lipopolysaccharide (LPS)–receptor CD14 and the Fcγ receptor CD16: classical (CD14++CD16−), non-classical (CD14+CD16++), and intermediate (CD14++CD16+) monocytes (8). Intermediate subsets are predictive of cardiovascular diseases (8) and are associated with susceptibility to plaque vulnerability (7), for example. Elevated levels of CD16+ subsets have been observed in patients with AAA (9), and a recent study showed that only intermediate monocytes (CD14++CD16+) were elevated in AAA patients compared to healthy individuals and peripheral artery disease (PAD) patients (10). Interestingly, elevated amounts of CD14++CD16+ and D-dimer proved to be suitable diagnostic markers for AAA and were able to predict rapidly growing AAA (10). However, it is still being debated whether the three subgroups are correlated to the main morphologic features of AAA—AAA diameter, AAA volume, and thickness of the intraluminal thrombus (ILT)—and whether they could be useful surrogate markers. Furthermore, it is unclear whether any of these monocyte subsets is associated with end-stage AAA (diameter > 50 mm), at which point most patients are diagnosed. The importance of immune mechanisms and emerging biomarkers has recently been demonstrated in patients with AAA (11, 12). The aim of this study was to comprehensively define circulating monocyte subsets and their associations with AAA risk and AAA morphology in an exploratory study.

## 2 Materials and methods

### 2.1 Study design

**Figure 1:**
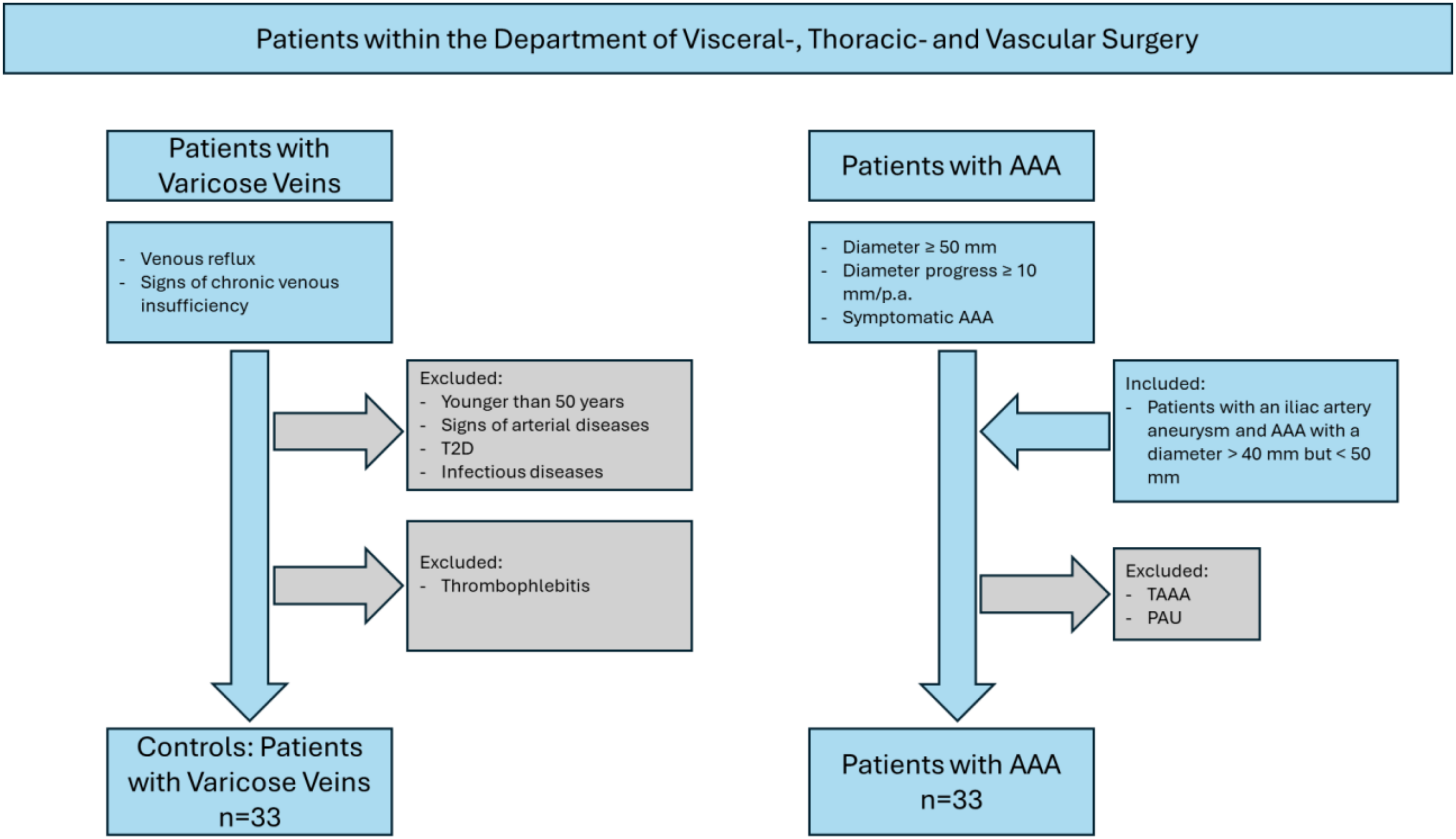
Schematic overview of the study design. T2D, type 2 diabetes mellitus; TAAA, thoracic abdominal aortic aneurysm; PAU, penetrating aortic ulcer.

An overview of the study design is depicted in **Figure 1**. Monocyte subpopulations were determined in an observational study in electively treated AAA patients and patients with varicose veins who served as controls. Patients with varicose veins were chosen as the control group because they had no evidence of cardiovascular diseases (4) in this group. The aim was to compare patients with AAA with non-diseased controls who had a normal aortic diameter. A similar control group was used in previous studies (13, 14). Due to the observational nature of the study, no sample size or power calculation was performed according to the strengthening the reporting of observational studies in epidemiology (STROBE) guidelines (15). Patients were treated in the Department of Visceral, Thoracic and Vascular Surgery between December 2020 and June 2024. The diagnostic criteria for varicose patients included venous reflux assessed using duplex ultrasound and signs of chronic venous insufficiency according to the Clinical, Etiological, Anatomical, and Pathophysiological (CEAP) classification (16). Inclusion criteria were age over 50 years and no known history of arterial disease, type 2 diabetes mellitus (T2D), and infectious diseases. Patients with thrombophlebitis were excluded. Inclusion criteria for AAA patients were a diameter of the infrarenal aorta of more than 50 mm, a rapidly growing AAA with more than 10-mm progression per year, or a symptomatic AAA. Two patients who were included were treated for rapidly growing iliac artery aneurysm but also showed an aortic dilation with a diameter >40 but <50 mm. The studies involving humans were approved by the Ethikkommission an der Technischen Universität Dresden (EK 151042017). The studies were conducted in accordance with the local legislation and institutional requirements. The participants provided written informed consent to participate in this study.

### 2.2 Outcome variables

Counts of each monocyte subset were defined as the primary outcome variable in the multivariable regression, while the presence or absence of AAA disease was defined as the outcome variable in the odds ratio (OR) analysis.

### 2.3 Clinical parameters

All blood parameters were measured in a non-fasting state. Serum low-density (LDL) and high-density lipoprotein (HDL), total cholesterol (TC), triglycerides, glucose, hemoglobin (Hb), leukocyte, and C-reactive protein (CRP) concentrations were analyzed in the Institute for Clinical Chemistry and Laboratory Medicine at TU Dresden using standard laboratory methods. Due to the lack of data on blood chemistry or medical therapies, the number of analyzed patients varied in each group. Cardiovascular risk factors, comorbidities, and prescribed medical therapies were evaluated retrospectively. Hypertension, T2D, heart failure (HF), PAD, and carotid artery stenosis (CAS) were defined using a past documented history of diagnosis or any treatment for these diseases. Coronary artery disease (CAD) was defined using a history of myocardial infarction, angina, or treatment for CAD. Smoking was defined using any history of smoking, and sex was self-reported.

### 2.4 Analysis of AAA diameter and AAA morphology

The aortic diameter, maximal intraluminal thrombus depth, and aortic volumes were determined as described previously (17, 18). In brief, aortic diameter was assessed using computed tomography angiography by a single trained observer by measuring the distance between the outer adventitia (outer-to-outer-edge). The thickness of the ILT was determined in the arterial phase using computed tomography (CT) scans following multiplanar reconstruction. The aorta was scanned in an axial position in 1-mm sections. The thickness of the ILT was assessed at the largest distance from the inner surface of the lumen to the outer aortic wall. The AAA volume was measured by two trained people using the automatic segmentation model of the IMPAX EE R20 software (Agfa HealthCare, Mortsel, Belgium). The aorta was scanned using contrast media in the arterial phase in 1-mm sections. The outer wall of the aneurysm and the true lumen were selected manually every 6 mm in the transverse plane. The starting point of the measurement was defined as the cranial end of the aneurysm with a diameter greater than 30 mm. The measurement ended caudal to the dilation or at the aortic bifurcation. Non-recognized areas were cropped manually. The volume of AAA is given in cm^3^. The ILT was included in the total AAA volume measurement but was not specifically tagged for segmentation.

### 2.5 Blood samples and flow cytometry

**Figure 2:**
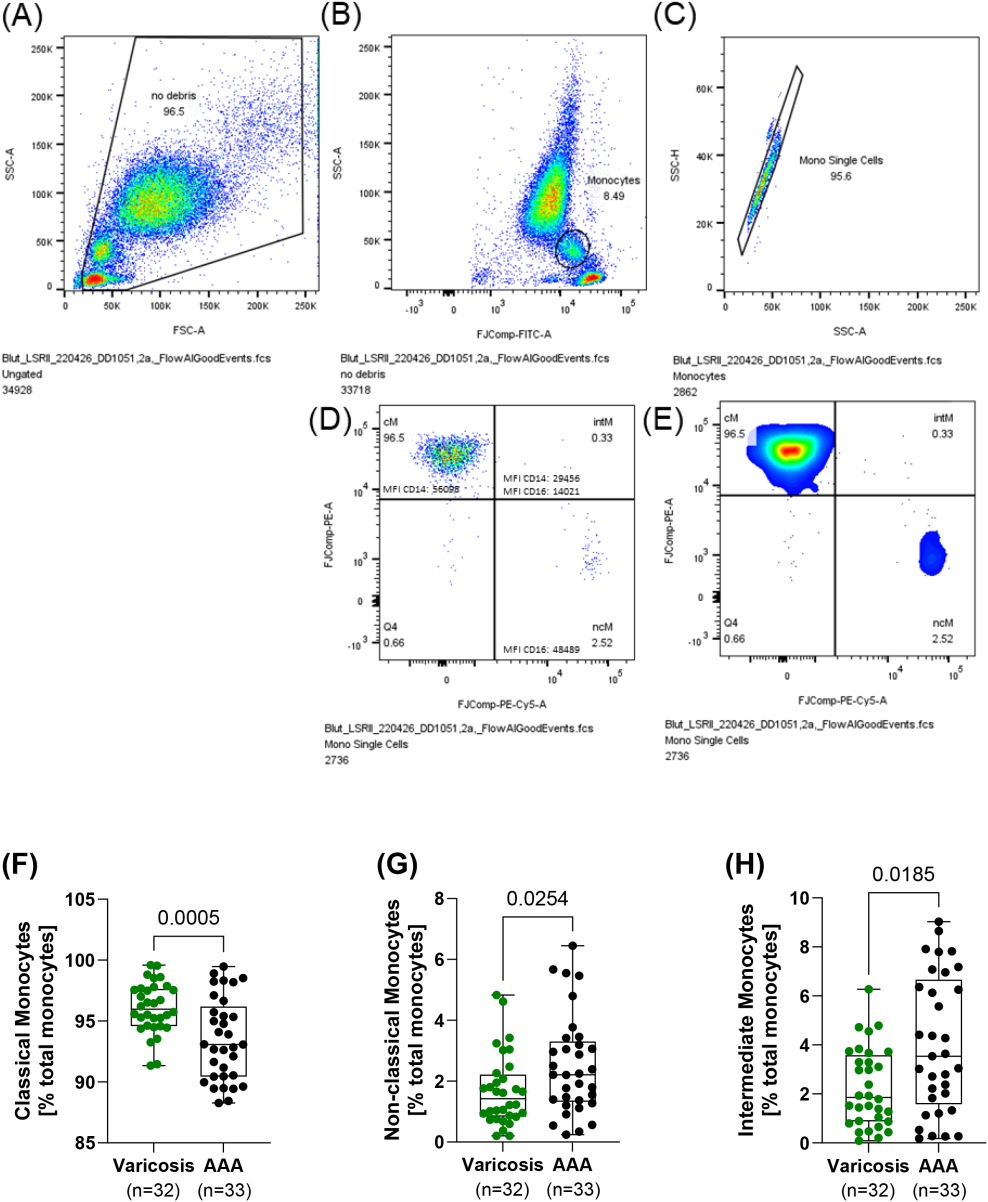
Monocyte subsets in patients with AAA and varicose veins. Non-fasting venous blood was collected, and the relative frequency of the individual monocyte subpopulations was determined using flow cytometry. Gating strategy: blood was collected and stained with CD45-FITC, CD16-PerCP710, and CD14-PE. **(A)** First, debris was excluded, and **(B)** in the next step, monocyte subpopulations were identified using CD45 expression and the SSC-A signal. **(C)** Cell aggregates were excluded from further analysis, and **(D, E)** monocyte subsets were determined regarding their expression of CD14 and CD16. **(F)** Classical (CD14++CD16−), **(G)** non-classical (CD14+CD16++), and **(H)** intermediate (CD14++CD16+) monocytes. All data are shown as box plots with individual values. Significant outliers were identified using Grubb’s outlier test, and one patient in the varicosis group was excluded from the analysis. **(F)** Unpaired t-test and **(G, H)** Mann–Whitney U test. AAA, abdominal aortic aneurysm.

Non‐fasting venous blood was collected pre-operatively between 2020 and 2024 in a 9 mL S-Monovette EDTA (Sarstedt, Nümbrecht, Germany). Monocyte subpopulations were analyzed using flow cytometry immediately after blood collection. Indeed, cells were stained for 10 min using the following antibody cocktail: CD45-FITC (HI-30, Thermo Fisher Scientific, Darmstadt, Germany), CD16-PerCP710 (CB16, Thermo Fisher Scientific, Darmstadt, Germany), and CD14-PE (MφP9, BD Biosciences, Heidelberg, Germany). Isotype controls were prepared to ensure the specificity of the antibodies. Afterward, erythrocytes were lysed by the addition of FACS lysing solution (BD Biosciences, Heidelberg, Germany) for 10 min. Data acquisition was performed on LSRFortessa (BD Biosciences, Heidelberg, Germany) and LSRII (BD Biosciences, Heidelberg, Germany). Data analysis was conducted using the FlowJo™ software (Version 10, BD Biosciences, Heidelberg, Germany). First, debris was excluded by applying side scatter (SSC) and forward scatter (FSC) signals, and events with low FSC and SSC signals were omitted. Before cell aggregates were excluded from the analysis, the expression of CD45 against the SSC signal was used to select the monocyte population for further analysis. Finally, classical (CD14++CD16−), non-classical (CD14+CD16++), and intermediate (CD14++CD16+) monocytes were determined according to their expression of CD14 and CD16. The percentage of monocytes is given relative to the total number of monocytes (**Figure 2**).

### 2.6 Statistics

GraphPad Prism 10.0 (GraphPad Software, Inc., La Jolla, CA, USA) and R Core Team (2021) R: A Language and Environment for Statistical Computing software (R Foundation for Statistical Computing, Vienna, Austria) were used for statistical analysis. A *p*-value ≤0.05 was considered significant. Significant outliers were identified using Grubb’s outlier test and normality distribution with the D’Agostino and Pearson test. According to the result of the normal distribution test, the Mann–Whitney U or unpaired t-test was used to compare AAA and varicose vein patients. In addition, the standardized mean difference (SMD) was calculated. Data are shown as box plots with individual values or as scatter dot plots, as indicated in the figure legends. Correlational analysis was conducted using Pearson’s correlation (r_P_). Penalized linear regression was performed for variable selection to assess the influence of age, sex, smoking, hypertension, T2D, CAD, PAD, CRP, HDL cholesterol, LDL cholesterol, triglyceride, hemoglobin, leukocyte count, AAA diameter, thickness of the ILT, and prescription of insulin, diuretics, statins, angiotensin-converting enzyme (ACE) inhibitors, calcium channel blocker (CCB), angiotensin II receptor blockers (ARBs), beta (β)-blocker, acetylsalicylic acid (ASA), and therapeutic anticoagulation. Data were analyzed by multivariable regression using each of the log-transformed monocyte counts as an independent variable for the presence of AAA disease. The effect of risk factors was compared with patients in whom the risk factor was not present. As the cohorts strongly differed in terms of age and sex, these two factors were included in this analysis. In addition, it was found that the comorbidities confounded each other. For this reason, and since patients with PAD had already served as the control group in previous studies (10), PAD was selected for inclusion in the multivariable analysis.

## 3 Results

### 3.1 Patient characteristics

A comparison of patients with AAA and patients with varicose veins revealed that the AAA group was significantly older and comprised a higher proportion of male patients. As expected, the two groups differed in terms of the prevalence of cardiovascular risk factors and prescribed pharmacotherapy. A detailed list of these risk factors and pharmacotherapy is provided in **Table 1**.

**Table 1:** Clinical characteristics in patients with abdominal aortic aneurysm (AAA) and varicose veins.

Observational study						
	Varicosis	AAA	*p*-Value	Statistics	Degrees of freedom	Standardized mean difference
Baseline demographics						
n included	33	33				
Age [years] mean ± SD	62.79 ± 8.15	70.29 ± 9.41	<0.001^a^	t = 3.51	64	0.86
Sex, male:female, % male	20:13, 61	30:3, 91	0.0041^c^	χ^2^ = 8.25	1	
Hematology						
Hemoglobin [mmol/L] mean ± SD, n	8.99 ± 0.73 32	9.01 ± 1.10 33	0.948^a^	t = 0.07	64	0.02
Reference values 8.60–12.10 mmol/L						
Leukocytes [GPt/L] mean ± SD, n	6.83 ± 1.48 32	7.64 ± 1.68 33	0.046^a^	t = 2.04	63	0.51
Reference values 3.8–9.8 GPt/L						
Cardiovascular risk factors						
LDL cholesterol—mmol/L, mean ± SD, n	4.18 ± 1.50 31	3.28 ± 1.36 33	0.014^a^	t = 2.53	62	0.63
Reference values <1.40 mmol/L for people with very high risk						
HDL cholesterol—mmol/L, median with range, n	1.63 (1.00–2.62) 31	1.28 (0.75–1.98) 33	0.015^b^	U = 331.00		0.71
Reference values >0.90 mmol/L for men and >1.10 mmol/L for women						
Total cholesterol—mmol/L, mean ± SD, n	4.57 ± 1.37 31	3.52 ± 1.55 33	0.006^a^	t = 0.85	62	0.71
Reference values <4.00 mmol/L						
Triglycerides—mmol/L, median with range, n	1.18 (0.43–3.88) 31	1.20 (0.52–3.59) 32	0.582^b^	U = 455.50		0.11
Reference values 0.35–1.70 mmol/L						
Blood glucose—mmol/L, median with range, n	5.37 (4.30–6.81) 28	5.94 (4.69–11.33) 30	0.004^b^	U = 238.50		0.84
Reference value only for fasting glucose is possible						
CRP—mg/L, median with range, n	1.30 (0.50–21.30) 31	2.65 (0.60–16.80) 31	0.049^b^	U = 353.50		0.20
Reference values <5.0 mg/L						
Smoking, yes:no—%	6:27, 18	23:10, 70	<0.001^c^	χ^2^ = 17.78	1	
Hypertension, yes:no—%	11:22, 33	30:3, 91	<0.001^c^	χ^2^ = 23.24	1	
CAD, yes:no—%	0:33, 0	10:23, 30	0.001^c^	χ^2^ = 11.79	1	
HF, yes:no—%	1:32, 3	10:23, 30	0.003^c^	χ^2^ = 8.84	1	
CAS, yes:no—%	0:33, 0	7:26, 21	0.005^c^	χ^2^ = 7.83	1	
PAD, yes:no—%	0:33, 0	8:25, 24	0.003^c^	χ^2^ = 9.10	1	
T2D, yes:no—%	0:33, 0	11:22, 33	<0.001^c^	χ^2^ = 13.20	1	
BMI—kg/m², median with range, n	27.20 (21.20–40.70) 33	27.25 (20.30–38.01) 33	0.677^b^	U = 511.50		0.14
Pharmacological therapies						
Statins, yes:no—%	3:29, 9	24:9, 73	<0.001^c^	χ^2^ = 26.85	1	
ACE inhibitors, yes:no—%	3:29, 9	15:18, 45	0.001^c^	χ^2^ = 10.56	1	
ARB, yes:no—%	4:28, 13	13:20, 39	0.014^c^	χ^2^ = 6.08	1	
CCB, yes:no—%	2:30, 6	13:20, 39	0.002^c^	χ^2^ = 10.05	1	
ASA, yes:no—%	2:30, 6	25:8, 76	<0.001^c^	χ^2^ = 32.32	1	
β-Blocker, yes:no—%	4:28, 13	17:16, 52	<0.001^c^	χ^2^ = 11.31	1	
Anticoagulation, yes:no—%	6:26, 19	3:30, 9	0.260^c^	χ^2^ = 1.27	1	
Antiplatelet, yes:no—%	1:31, 3	3:30, 9	0.317^c^	χ^2^ = 1.00	1	
Diuretics, yes:no—%	4:28, 13	9:24, 27	0.137^c^	χ^2^ = 1.49	1	
T2D treatment, yes:no—%	0:32, 0	11:22, 33	<0.001^c^	χ^2^ = 12.84	1	
Insulin, yes:no—%	0:32, 0	2:31, 6	0.157^c^	χ^2^ = 2.00	1	

Blood was taken in the non-fasted state. Reference values for the corresponding blood parameters are given in the table. Reference values for LDL and total cholesterol (TC) are based on the European Society of Cardiology (ESC) recommendations. Reference values for fasting glucose were excluded due to withdrawal in the non-fasted state. Statistics: All data are presented as median with minimum and maximum values. Comparison of continuous data was conducted using the Mann–Whitney U test or t-test. Discrete data were analyzed by the chi-square test. For t test (a), t-statistic is reported; Mann–Whitney (b), U statistics; and chi-square (c), χ^2^.

ACE, angiotensin-converting enzyme; ARB, angiotensin II receptor blocker; BMI, body mass index; ASA, acetylsalicylic acid; CAD, coronary artery disease; CCB, calcium channel blocker; HDL, high-density lipoprotein; LDL, low-density lipoprotein; PAD, peripheral artery disease; T2D, type 2 diabetes mellitus; TIA, transient ischemic attack.

### 3.2 Phenotypic heterogeneity of monocyte subsets in AAA patients

In the present study, significant shifts in monocyte subsets were observed in AAA patients compared to controls with varicose veins. Patients with AAA had a 2.6% lower proportion of classical monocytes (AAA: 93.5% ± 3.4% *vs*. controls: 96.1% ± 2.0%, *p* = 0.001, SMD = 0.91). Conversely, patients with AAA exhibited a 1.9-fold increase in non-classical monocytes (AAA: 2.5% ± 1.6% *vs*. controls: 1.7% ± 1.2%, *p* = 0.025, SMD = 0.58) and a 1.8-fold higher proportion of intermediate monocytes (AAA: 4% ± 2.8% *vs*. controls: 2.3% ± 1.6%, *p* = 0.019, SMD = 0.77) (**Figure 2**). A linear weighted regression was performed to determine which of the patients’ risk factors and comorbidities had the most influence on the subgroups. We found that classical monocytes were relatively robust since they were barely affected by any of the investigated comorbidities or risk factors (**Supplementary Table 1**). In non-classical monocytes, PAD was associated with an increase in comorbidities or risk factors, while T2D and hypertension were associated with a decrease in this subset (**Supplementary Table 2**). Intermediate monocytes were strongly influenced by the presence of T2D, while sex and hypertension were associated with a decrease in this subset (**Supplementary Table 3**). All of the comorbidities and risk factors investigated were found to confound each other to a high degree. Additionally, there were the greatest differences in age and sex between the AAA and control groups. Therefore, we chose age, sex, and PAD for the multivariable regression analysis to analyze whether these factors influence the observed differences between patients with varicose veins and patients with AAA. After adjusting for age, sex, and PAD, the observed decrease in classical monocytes (*p* = 0.001) and increase in non-classical monocytes (*p* = 0.040) in AAA patients remained significant (**Table 2**).

**Table 2:** Differences in monocyte subsets after adjusting for age, sex, and PAD.

Variable	Unadjusted OR [95% CI]	*p*-Value	Adjusted OR [95% CI]	*p*-Value
Classical monocytes [log10]				
Group (ref = varicosis)	0.973 [0.958, 0.987]	<0.001	0.968 [0.949, 0.987]	0.001
Age (years)	0.999 [0.998, 1.000]	0.076	1.000 [0.999, 1.001]	0.950
Sex (ref = female)	1.001 [0.982, 1.021]	0.895	1.014 [0.994, 1.035]	0.160
PAD (ref = none)	0.987 [0.963, 1.012]	0.308	1.004 [0.980, 1.029]	0.754
Non-classical monocytes [log10]				
Group (ref = varicosis)	1.504 [1.019, 2.220]	0.040	1.760 [1.058, 2.928]	0.030
Age (years)	1.002 [0.981, 1.024]	0.830	0.988 [0.964, 1.012]	0.331
Sex (ref = female)	1.024 [0.635, 1.652]	0.921	0.784 [0.462, 1.330]	0.360
PAD (ref = none)	1.283 [0.697, 2.360]	0.417	1.026 [0.537, 1.960]	0.937
Intermediate monocytes [log10]				
Group (ref = varicosis)	1.674 [0.988, 2.836]	0.055	1.940 [0.987, 3.814]	0.054
Age (years)	1.024 [0.996, 1.053]	0.091	1.007 [0.975, 1.040]	0.680
Sex (ref = female)	0.709 [0.375, 1.343]	0.287	0.563 [0.279, 1.136]	0.107
PAD (ref = none)	1.203 [0.527, 2.745]	0.656	0.857 [0.363, 2.024]	0.721

Monocyte subsets were analyzed using flow cytometry, and monocytes are given in % of total monocytes. Data were analyzed by multivariable regression using each of the monocyte subgroups as a diagnostic variable for the presence of AAA disease. The monocyte counts were logarithmically transformed, and data were analyzed by multivariable linear regression using the corresponding subset as a predictor variable. The effects of each risk factor were compared with patients in whom the risk factor was not present (ref = none). The odds ratio (OR) refers to the relative increase in risk of AAA disease as the percentage of the subgroup increases. For sex, OR refers to men, and reference values refer to women. AAA diameter and age show the increase per unit (mm and year, respectively). Unadjusted values were obtained by pairwise comparison of each variable listed in the table with the outcome of having an AAA. Adjusted values were analyzed by holding the effects of the other variables constant and assuming an increase in the monocyte counts on the logarithmic scale.

OR, odds ratio; PAD, peripheral artery disease; AAA, abdominal aortic aneurysm.

### 3.3 Monocyte subsets and associations with AAA morphology and AAA risk

ORs were calculated to test whether differences in subgroups were associated with an increased risk of AAA disease. A reduced number of classical monocytes was associated with an increased risk of AAA (*p* = 0.002). Furthermore, higher levels of non-classical (*p* = 0.030) and intermediate monocytes (*p* = 0.006) were found to be associated with an increased risk of AAA (**Table 3**).

**Table 3:** Odds ratios of monocyte subsets and AAA risk.

	Odds ratio [95% CI]	*p*-Value
Classical monocytes	0.726 [0.584, 0.874]	0.002
Intermediate monocytes	1.412 [1.123, 1.846]	0.006
Non-classical monocytes	1.537 [1.068, 2.354]	0.030

Monocyte subsets were analyzed using flow cytometry and are given in % of total monocytes. Logistic regression was used to obtain the odds ratio of incident AAA per one-unit increase in monocyte counts on a log scale.

**Figure 3:**
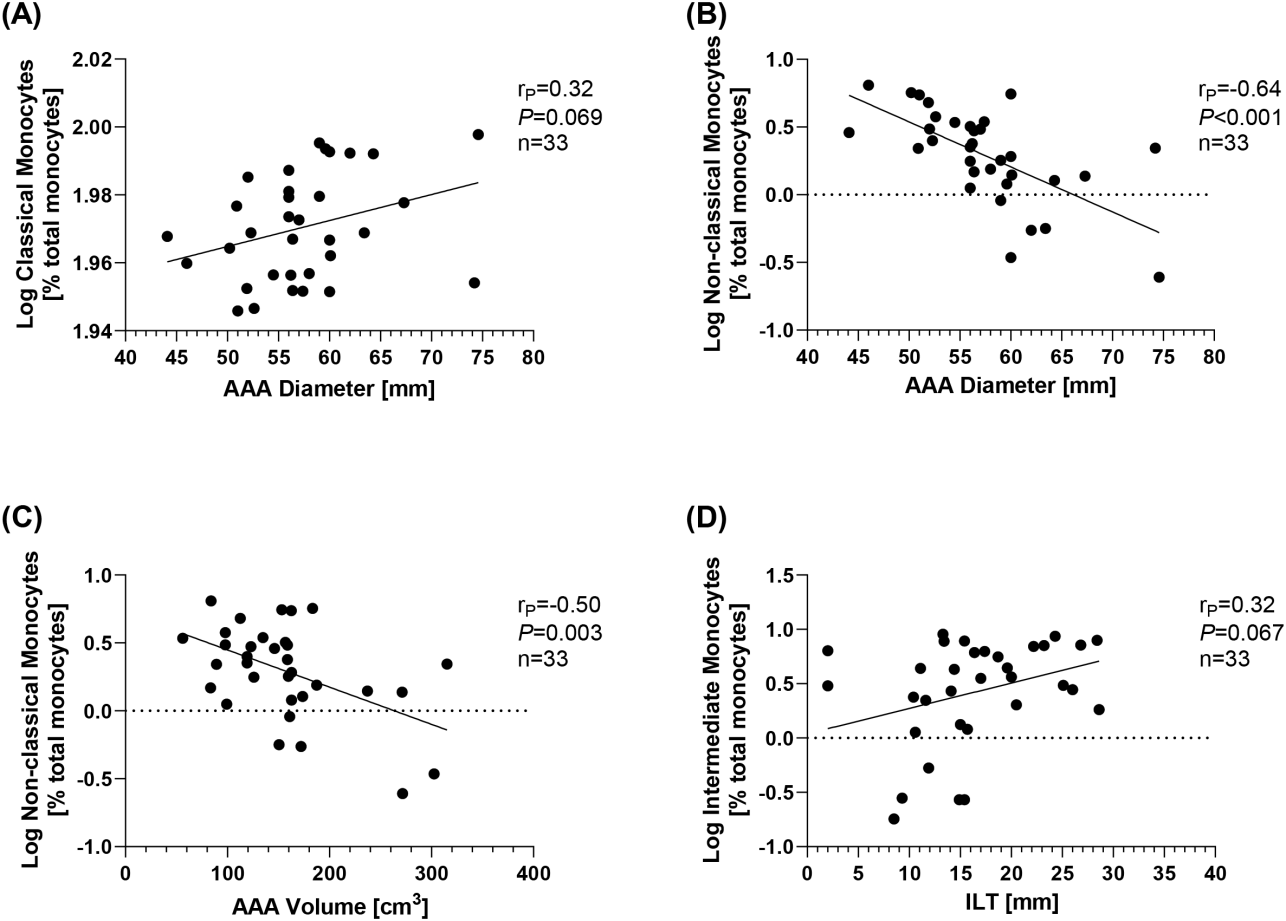
Correlations of monocyte subsets and AAA morphology. Monocyte subsets were analyzed using flow cytometry and are given as % total monocytes. Data were log-transformed and analyzed for correlations with features of AAA morphology. Correlation of **(A)** classical monocytes and AAA diameter, **(B)** non-classical monocytes and AAA diameter, **(C)** non-classical monocytes and AAA volume, and **(D)** intermediate monocytes and ILT. Statistics: Grubb’s outlier test was used to identify outliers. One AAA patient was identified as an outlier and excluded from the analysis. Correlational analysis was conducted using Pearson’s correlation. r_P_, Pearson’s rank correlation coefficient; AAA, abdominal aortic aneurysm; ILT, thickness of the intraluminal thrombus.

A correlational analysis was performed to test whether the different monocyte subgroups are associated with AAA morphology and could therefore serve as surrogate markers. There was a trend toward an increase in classical monocytes with AAA diameter (r_P_ = 0.32, *p* = 0.069) (**Figure 3A**), although no correlations were found with AAA volume or ILT thickness (**Table 4**). Conversely, non-classical monocytes decreased with increasing AAA diameter (r_P_ = −0.64, *p* < 0.001) and increasing AAA volume (r_P_ = −0.50, *p* = 0.003) (**Figures 3B, C**). Intermediate monocytes showed a trend toward a positive association with the maximum ILT thickness (r_P_ = 0.32, *p* = 0.067) (**Figure 3D**). **Table 4** provides an overview of all correlations tested.

**Table 4:** Summary of correlations between monocyte subsets and AAA morphology.

	Classical monocytes		Non-classical monocytes		Intermediate monocytes	
	r_P_	*p*-Value	r_P_	*p*-Value	r_P_	*p*-Value
**AAA diameter**	0.32	0.069	**−0.64**	**<0.001**	−0.20	0.265
**ILT**	−0.21	0.23	0.19	0.298	**0.32**	**0.067**
**AAA volume**	0.27	0.13	**−0.50**	**0.003**	−0.13	0.481

Monocyte subsets were analyzed using flow cytometry and are given in % of total monocytes. Data were log-transformed. Correlational analysis was performed using Pearson’s correlation. Significant outliers were identified using Grubb’s outlier test and excluded from the analysis.

AAA, abdominal aortic aneurysm; ILT, intraluminal thrombus; r_P_, Pearson’s rank correlation coefficient. The numbers marked in bold in the table indicate significant associations or trends in these.

## 4 Discussion

Monocytes play a role in the pathology of AAA and can exhibit immunophenotypic heterogeneity. While alterations in monocyte subsets are associated with cardiovascular risk, their profile and associations with AAA diameter and AAA morphology remain poorly understood. We could demonstrate that the number of classical monocytes was lower in AAA, whereas the number of intermediate and non-classical monocytes was higher. Interestingly, these changes were associated with an increased risk of AAA. Furthermore, there was an inverse correlation between non-classical monocytes and AAA diameter and volume.

The present study revealed lower counts of classical monocytes (CD14++CD16−) and higher counts of intermediate (CD14++CD16+) and non-classical monocytes (CD14+CD16++). Our data are, at least in part, supported by previously published studies (9, 10). The decreased proportion of classical monocytes could be explained by findings in mice following the induction of acute myocardial infarction. In the first phase, CD14++CD16− monocytes accumulated at the site of injury early on to clear necrotic debris and promote the healing of the damaged tissue. In the second phase, CD14+CD16++ non-classical monocytes dominated and promoted healing by stimulating the accumulation of myofibroblasts, angiogenesis, and collagen deposition (19). The data herein eventually underline an imbalance between both subsets in patients with AAA, with intermediate and non-classical subsets being more prominent. Classical monocytes can transmigrate through the endothelium into the vessel wall (20). This could explain the depletion of the classical monocytes, which may be due to an increased migration into the AAA.

Under both physiological and pathological conditions, non-classical monocytes actively patrol the vascular endothelium (21). They have phagocytic properties, enabling them to remove cell debris and play an important role in controlling vascular integrity. They are most likely involved in the resolution of inflammation (21). Studies in patients with CAD (22) and subclinical atherosclerosis demonstrated (23) increased non-classical subsets and therefore coincide with the data of the present study. Here, a higher count of non-classical monocytes was found in AAA patients, which differs from the data previously published by Klopf et al., who used PAD patients as a control group (10). Patients with PAD are most often used as controls in studies of AAA (10, 17). PAD is one of the most common initial manifestations of T2D (24), and T2D itself is inversely associated with AAA disease (25). It could be speculated that confounding of the protective and adverse effects could occur. The increase in intermediate and non-classical monocyte subsets could be further explained at the expense of classical monocytes. Similar results have been demonstrated in patients with T2D (26). In support of this hypothesis, classical monocytes differentiate into non-classical monocytes via intermediate monocytes (27). It has been shown that intermediate monocytes are inflammatory and are an independent predictor of cardiovascular events in patients with atherosclerotic diseases (8). A similar increase was observed in patients with advanced PAD, indicating disease progression (28).

To further understand the link between AAA risk and blood monocytes, we analyzed whether a specific subtype of monocytes was associated with an increased risk of having AAA disease. We were able to show that a lower proportion of classical monocytes was associated with an increased risk of AAA, while an increase in non-classical and intermediate monocytes was also associated with an increased risk of AAA. In line with this, it has previously been shown that the percentage of classical monocytes is decreased in PAD patients in the advanced state (28). Classical monocytes secrete cytokines and are known to be phagocytic (29), are activated by inflammatory cues, and act during the acute phase of inflammation (29). As with other cardiovascular diseases, AAA could be considered a chronic inflammatory condition (30) rather than an acute infection.

Analyzing AAA volume has the advantage of taking into account the morphology of the aneurysm (31). AAA volume can be useful when diameter measurement is not possible, for example, in monitoring saccular AAA, since there is a weaker correlation between increased diameter and rupture risk in this type of AAA (32). Finally, AAA volume may be a better predictor of AAA growth (33). The present study demonstrated that an increased proportion of non-classical (CD14+CD16++) monocytes was found to be inversely associated with AAA diameter and AAA volume. A negative correlation was found between non-classical (CD14+CD16++) monocytes and intima–media thickness in patients with carotid artery stenosis (34) and PAD (28). The severity of these diseases was associated with a decrease in non-classical subsets (28), which is consistent with the findings of the present study. Non-classical (CD14+CD16++) monocytes (or Ly-6C^lo^ in mice) are involved in the healing of the ischemic myocardium, initiating differentiation into anti-inflammatory M_2_ macrophages, which are known to be involved in tissue repair (19, 35). It is speculative, but the inverse relationship could be explained by temporal patterns and a lack of their protective and healing functions (34), although non-classical subsets are higher in patients with AAA. Additionally, the patients examined here were in the final stage of the disease and exhibited typical AAA wall degeneration (17, 36). Another possibility that could explain the inverse relationship between AAA diameter and non-classical monocytes is that, as the diameter increases, more non-classical monocytes migrate into the AAA, and their concentration in the blood decreases. The migration of non-classical monocytes is strongly context-dependent and has been observed in other cardiovascular diseases (37).

The majority of AAAs is covered by the ILT. The ILT contains fibrin, inflammatory cells, platelets, and red blood cells and affects the growth and rupture of the AAA (38). Herein, intermediate (CD14++CD16+) subsets tended to correlate with ILT thickness. It has been demonstrated that intermediate monocytes are present in the ILTs, albeit in lower amounts than in the peripheral blood of AAA patients (39). The positive association could reflect the inflammatory nature of the ILT or the inflammatory intermediate monocyte subset (8).

Overall, the present study revealed age- and sex-independent shifts in the classical, non-classical, and intermediate subsets in AAA, suggesting disease-specific mechanisms. Decreases in classical monocytes and increases in intermediate and non-classical monocytes were associated with an increased risk of AAA. The number of non-classical monocytes correlated inversely with AAA diameter and volume, suggesting they could be useful surrogate markers for AAA morphology beyond the AAA diameter. The decrease in classical monocytes and the increase in non-classical monocytes should be especially considered in future studies.

## 5 Limitations

This study is descriptive, explorative, and observational, based on a very small cohort of patients. Patients with varicose veins were used as the control group, but they differ from the AAA group in terms of age, comorbidity, and risk factor profile. Furthermore, varicose veins are reported to be either an inflammatory disease or affected by inflammation. Moreover, our analysis was limited to patients who met the surgical criteria for AAA repair (diameter > 50 mm). We did not include patients with small AAA (30–50 mm), as these are important for drug development because they represent the early stages of the disease. The relatively small number of patients (n = 33) included, coupled with the large number of confounding factors, may have influenced the results. We cannot exclude that other variables, such as medical therapies or cardiovascular risk factors, may have affected the results. Analyzing patients by adjusting their risk factors in advance, for example, in a case–control study, could minimize these effects. Furthermore, the results obtained here permit the establishment of a causal relationship between the different monocyte subsets and the development of AAA due to the study design. Finally, the main discrepancy when comparing data from flow cytometry with those from the present study is the pre-selection of cells, the declaration of subsets based on CD14 and CD16 expression, and the gating strategy in flow cytometry.

## Data Availability

The original contributions presented in the study are included in the article/**Supplementary Material**. Further inquiries can be directed to the corresponding author.
