# Supplementary material for: Changes in monocyte subsets are associated with an increased risk of AAA and are surrogate markers for AAA morphology in patients with late-stage disease
Source: Front Immunol. 2025 Sep 3;16:1621888. doi: 10.3389/fimmu.2025.1621888 (PMC12442832; doi:10.3389/fimmu.2025.1621888)
Supplement: Supplementary file 2 [file DataSheet2.docx]

**Supplementary Material**

**Changes in monocyte subsets are associated with an increased risk of AAA and are surrogate markers for AAA morphology in patients with late-stage disease**

**Short title:** Monocyte subsets in patients with AAA

Bianca Hamann^1^, Anna Klimova^2^, Marvin Kapalla^1^, David M. Poitz^3^, Albert Busch^1^, Henning Morawietz^4^, Christian Reeps^1^† and Anja Hofmann^1^†*****

†These authors share senior authorship

^1^Division of Vascular and Endovascular Surgery, Department of Visceral, Thoracic and Vascular Surgery, University Hospital and Medical Faculty Carl Gustav Carus, TUD Dresden University of Technology, Germany;

^2^Institute for Medical Informatics and Biometry, Faculty of Medicine, TUD Dresden University of Technology, Germany;

^3^Institute for Clinical Chemistry and Laboratory Medicine; University Hospital and Medical Faculty Carl Gustav Carus, TUD Dresden University of Technology, Germany;

^4^Division of Vascular Endothelium and Microcirculation, Department of Medicine III, University Hospital and Medical Faculty Carl Gustav Carus, TUD Dresden University of Technology, Germany;

***Corresponding author:**

Anja Hofmann, PhD

Division of Vascular and Endovascular Surgery

Department of Visceral‐, Thoracic and Vascular Surgery

Faculty of Medicine and University Hospital Carl Gustav Carus

Technische Universität Dresden

Fetscherstraße 74

D‐01307 Dresden, Germany

Email: anja.hofmann2@uniklinikum-dresden.de

**Supplemental Tables**

**Supplemental Table 1** **Classical monocytes in response to different cardiovascular risk factors in AAA.** The data were log-transformed and penalized linear regression was performed. Classical monocytes were determined by flow cytometry, and their number was set as the outcome variable. The effects of AAA diameter, ILT thickness, smoking, CHD, PAD, T2D, hypertension, BMI, gender, and age were analyzed using multivariable linear regression. Estimates show the increase or decrease in classical monocytes when the patient has the indicated disease or risk factor (ref = none). For AAA diameter, thickness of ILT, BMI, and age, values refer to the increase or decrease per one unit**. Abbreviations**: AAA, abdominal aortic aneurysm; BMI, body mass index; CAD, coronary artery disease; ILT, intraluminal thrombus; PAD, peripheral artery disease; T2D, type 2 diabetes mellitus.

| **Variable** | **Weight**  **estimates** |
| --- | --- |
| AAA diameter (mm) | 1.003 |
| Thickness ILT (mm) | 0.998 |
| Age (years) | 1.001 |
| Sex (ref = female) | 1.057 |
| BMI | 1.001 |
| smoking (ref = no) | 1.012 |
| PAD (ref = no) | 0.995 |
| CAD (ref = no) | 1.018 |
| T2D (ref = no) | 0.976 |
| Hypertension | 1.045 |

**Supplemental Table 2: Non-classical monocytes in response to different cardiovascular risk factors in AAA**. The data were log-transformed, and penalized linear regression was performed. Non-classical monocytes were determined by flow cytometry and defined as an outcome variable. The effects of AAA diameter, ILT thickness, smoking, CHD, PAD, T2D, hypertension, BMI, sex, and age were analyzed using multivariable linear regression. The estimates show the increase or decrease in non-classical monocytes if the patient has the specified disease or risk factor (ref = none). For AAA diameter, thickness of ILT, BMI, and age, values refer to the increase or decrease per one unit. **Abbreviations:** AAA, abdominal aortic aneurysm; BMI, body mass index; CAD, coronary artery disease; ILT, intraluminal thrombus; PAD, peripheral artery disease; T2D, type 2 diabetes mellitus.

| **Variable** | **Weight**  **estimates** |
| --- | --- |
| AAA diameter (mm) | 0.914 |
| Thickness ILT (mm) | 1.040 |
| Age (years) | 0.991 |
| Sex (ref = female) | 0.943 |
| BMI | 1.016 |
| smoking (ref = no) | 0.808 |
| PAD (ref = no) | 1.490 |
| CAD (ref = no) | 0.850 |
| T2D (ref = no) | 0.738 |
| Hypertension | 0.507 |

**Supplemental Table 3**: **Intermediate monocytes in response to different cardiovascular risk factors in AAA.** The data were log-transformed, and penalized linear regression was performed. Intermediate monocytes were determined by flow cytometry and defined as an outcome variable. The effects of AAA diameter, ILT thickness, smoking, CHD, PAD, T2D, hypertension, BMI, sex, and age were analyzed using multivariable linear regression. The estimates show the increase or decrease in intermediate monocytes if the patient has the indicated disease or risk factor (ref = none). For AAA diameter, thickness of ILT, BMI, and age, values refer to the increase or decrease per one unit. **Abbreviations**: AAA, abdominal aortic aneurysm; BMI, body mass index; CAD, coronary artery disease; ILT, intraluminal thrombus; PAD, peripheral artery disease; T2D, type 2 diabetes mellitus

| **Variable** | **Weight**  **estimates** |
| --- | --- |
| AAA diameter (mm) | 0.937 |
| Thickness ILT (mm) | 1.063 |
| Age (years) | 0.981 |
| Sex (ref = female) | 0.219 |
| BMI | 0.923 |
| smoking (ref = no) | 1.106 |
| PAD (ref = no) | 0.776 |
| CAD (ref = no) | 0.955 |
| T2D (ref = no) | 2.748 |
| Hypertension | 0.581 |
