# Supplementary material for: Changes in monocyte subsets are associated with an increased risk of AAA and are surrogate markers for AAA morphology in patients with late-stage disease
Source: Front Immunol. 2025 Sep 3;16:1621888. doi: 10.3389/fimmu.2025.1621888 (PMC12442832; doi:10.3389/fimmu.2025.1621888)
Supplement: Supplementary file 3 [file DataSheet3.docx]

**Supplementary Material**

**Supplemental Figures**


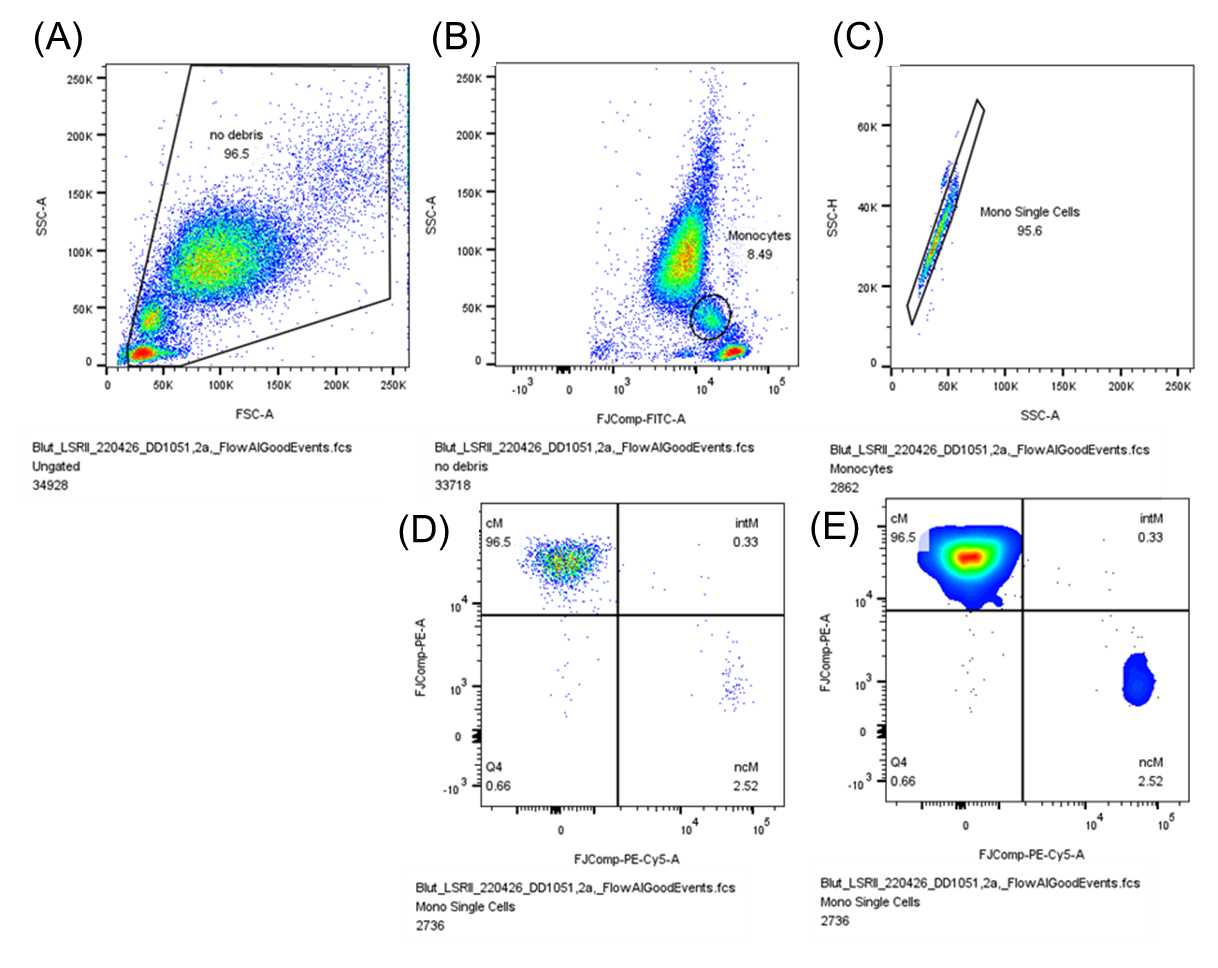


**Supplemental Figure 1** **Gating Strategy of Monocyte Subpopulations in Flow Cytometry.** Non-fasting venous blood was collected and stained with CD45-FITC, CD16-PerCP710 and CD14-PE. **(A),** First, debris was excluded and **(B),** in the next step, monocyte subpopulation were identified using CD45 expression and the SSC-A signal. **(C),** Cell aggregates were excluded from further analysis and **(D), (E)** monocyte subsets were determined regarding their expressions of CD14 and CD16.

**Supplemental Tables**

**Supplemental Table 1** **Classical monocytes in response to different cardiovascular risk factors in AAA.** The data were logarithmically transformed and weighted linear regression was performed. Classical monocytes were determined by flow cytometry, and their number was set as the outcome variable. The effects of AAA diameter, ILT thickness, smoking, CHD, PAD, T2D, hypertension, BMI, gender and age were analyzed using multivariate linear regression. Estimates show the increase or decrease in classical monocytes when the patient has the indicated disease or risk factor (ref = none). For AAA diameter, thickness of ILT, BMI and age, values refer to the increase or decrease per one unit**. Abbreviations**: AAA, abdominal aortic aneurysm; BMI, body mass index; CAD, coronary artery disease; ILT, intraluminal thrombus; PAD, peripheral artery disease; T2D, type 2 diabetes mellitus.

| **Variable** | **Estimates** |
| --- | --- |
| AAA diameter (mm) | 1.003 |
| Thickness ILT (mm) | 0.998 |
| Age (years) | 1.001 |
| Sex (ref = female) | 1.057 |
| BMI | 1.001 |
| smoking (ref = no) | 1.012 |
| PAD (ref = no) | 0.995 |
| CAD (ref = no) | 1.018 |
| T2D (ref = no) | 0.976 |
| Hypertension | 1.045 |

**Supplemental Table 2: Non-classical monocytes in response to different cardiovascular risk factors in AAA**. The data were logarithmically transformed and weighted linear regression was performed. Non-classical monocytes were determined by flow cytometry and defined as outcome variable. The effects of AAA diameter, ILT thickness, smoking, CHD, PAD, T2D, hypertension, BMI, sex, and age were analyzed using multivariate linear regression. The estimates show the increase or decrease in non-classical monocytes if the patient has the specified disease or risk factor (ref = none). For AAA diameter, thickness of ILT, BMI and age, values refer to the increase or decrease per one unit. **Abbreviations:** AAA, abdominal aortic aneurysm; BMI, body mass index; CAD, coronary artery disease; ILT, intraluminal thrombus; PAD, peripheral artery disease; T2D, type 2 diabetes mellitus.

| **Variable** | **Estimates** |
| --- | --- |
| AAA diameter (mm) | 0.914 |
| Thickness ILT (mm) | 1.040 |
| Age (years) | 0.991 |
| Sex (ref = female) | 0.943 |
| BMI | 1.016 |
| smoking (ref = no) | 0.808 |
| PAD (ref = no) | 1.490 |
| CAD (ref = no) | 0.850 |
| T2D (ref = no) | 0.738 |
| Hypertension | 0.507 |

**Supplemental Table 3**: **Intermediate monocytes in response to different cardiovascular risk factors in AAA.** The data were logarithmically transformed and weighted linear regression was performed. Intermediate monocytes were determined by flow cytometry and defined as outcome variable. The effects of AAA diameter, ILT thickness, smoking, CHD, PAD, T2D, hypertension, BMI, gender and age were analyzed using multivariate linear regression. The estimates show the increase or decrease in intermediate monocytes if the patient has the indicated disease or risk factor (ref = none). For AAA diameter, thickness of ILT, BMI and age, values refer to the increase or decrease per one unit. **Abbreviations**: AAA, abdominal aortic aneurysm; BMI, body mass index; CAD, coronary artery disease; ILT, intraluminal thrombus; PAD, peripheral artery disease; T2D, type 2 diabetes mellitus

| **Variable** | **Estimates** |
| --- | --- |
| AAA diameter (mm) | 0.937 |
| Thickness ILT (mm) | 1.063 |
| Age (years) | 0.981 |
| Sex (ref = female) | 0.219 |
| BMI | 0.923 |
| smoking (ref = no) | 1.106 |
| PAD (ref = no) | 0.776 |
| CAD (ref = no) | 0.955 |
| T2D (ref = no) | 2.748 |
| Hypertension | 0.581 |
